# Language distance modulates cognitive control in bilinguals

**DOI:** 10.1038/s41598-021-02973-x

**Published:** 2021-12-16

**Authors:** Narges Radman, Lea Jost, Setareh Dorood, Christian Mancini, Jean-Marie Annoni

**Affiliations:** 1 School of Cognitive Sciences, Institute for Research in Fundamental Sciences (IPM) Opposite the ARAJ, Artesh Highway, Aghdassieh, Tehran, 1956836484 Iran; 2 Department of Psychiatry, Roozbeh Hospital, Tehran University of Medical Sciences, Tehran, Iran; 3 Neurology Unit, Medicine Section, Laboratory for Cognitive and Neurological Science, Department of Neuroscience and Movement Science, Faculty of Science and Medicine, University of Fribourg, Fribourg, Switzerland

**Keywords:** Cognitive control, Language

## Abstract

Linguistic processes in the bilingual brain are partially shared across languages, and the degree of neural overlap between the languages is influenced by several factors, including the age of acquisition, relative language proficiency, and immersion. There is limited evidence on the role of linguistic distance on the performance of the language control as well as domain-general cognitive control systems. The present study aims at exploring whether being bilingual in close and distant language pairs (CLP and DLP) influences language control and domain-general cognitive processes. We recruited two groups of DLP (Persian–English) and CLP (French–English) bilinguals. Subjects performed language nonswitching and switching picture-naming tasks and a nonlinguistic switching task while EEG data were recorded. Behaviorally, CLP bilinguals showed a lower cognitive cost than DLP bilinguals, reflected in faster reaction times both in language switching (compared to nonswitching) and nonlinguistic switching. ERPs showed differential involvement of cognitive control regions between the CLP and DLP groups during linguistic switching vs. nonswitching at 450 to 515 ms poststimulus presentation. Moreover, there was a difference between CLP and DLP groups from 40 to 150 ms in the nonlinguistic task. Our electrophysiological results confirm a stronger involvement of language control and domain-general cognitive control regions in CLP bilinguals.

## Introduction

### Adaptive bilingual language control

The potential impact of bilingualism on brain organization concerns both language representation and language control. Certain evidences suggest that knowing two languages has a direct and reinforcing impact on language control and on the more general cognitive control system^1^. Historically, bilingual language monitoring has been proposed to rely on cognitive control, a cognitive domain strongly related to executive functions (EFs). In previous studies, it has been suggested that EFs control language selection and production in the first and second languages (L1 and L2)^2–5^. Language representations of both target and nontarget languages are coactivated and compete during lexical selection processes, which potentially leads to conflicts and interferences between languages, bringing up the idea of an involvement of inhibitory control (IC^6^) in language monitoring. In their adaptive bilingual language control model, Abutalebi and Green^6^ suggest that IC is needed to avoid interference when the dominant L1 competes for access while speaking the nondominant L2. As such, executive processes such as suppression of interfering cues and use of facilitator information in performing a new task^7^ can be fostered by bilingual experience. More recently, the effect of bilingualism on EF has led to controversies^8^. While several studies have provided evidence that bilingualism has a positive effect on various aspects of EF, other studies find only partial or nonexistent effects of bilingualism (Refs.^9–11^; for reviews on this controversial issue see also Refs.^8,12,13^).

The level of competition between the spoken languages of bilinguals depends on several factors, including language background, language mode^14^, L2 proficiency^15,16^, degree of language immersion^17^, and bilingual experience^18^. In addition to these biographical factors (proficiency and age of acquisition), the linguistic structural differences between languages also play a role not only in language representation but also in language control. Linguistic differences refer to the extent to which two languages have different vocabulary, syntactic structure, phonemes, spelling and pronunciation, orthography and writing systems^19^. Certain orthographic and phonological features are language specific (e.g., alphabets and phonologies do not necessarily overlap across different languages). Overall, research on the role of such linguistic factors in the brains of bilingual subjects is limited.

### The role of language similarity in language control and domain-general cognitive control

Studies using spatially-sensitive methods suggest that linguistic distance modulates language representations in bilinguals and multilinguals. An fMRI study investigating how language distance affects the brain network involved in L1 and L2 reading showed that the network is comparable when the two languages are similar in orthographic transparency, while additional neural resources in the left middle frontal gyrus are needed when the L2 is more opaque (i.e. less transparent orthography-to-phonology mapping) than the L1^20^. Such additional recruitment of the left middle frontal gyrus was also reported in a study with early bilinguals revealing larger involvement of such frontal areas during reading in the more opaque as compared to the transparent language^21^. There is also evidence from clinical studies showing that linguistic differences matter, leading for example to different transfer patterns in bilingual people with aphasia. Clinical data also show that linguistic differences can lead to different transfer patterns in bilingual people with aphasia^22^. Linguistic similarity facilitates cross-language generalization after language therapy, which argues for shared neural networks in closely related languages^23^. However, there is limited knowledge about the role of linguistic distance on the convergent or divergent representation of languages in the brains of bilinguals. The convergent view postulates that both languages activate similar neuronal regions. The overlap of brain regions involved in L1 and L2 processing increases with increasing proficiency. The divergent view, however, states that L1 and L2 activate different brain regions^24^. Data on the possible effect of linguistic distance on the involvement of the language control system in bilingual language processing are scarce and mixed^25,26^. De Bot^27^ suggests that the sharedness of the lexical systems of two languages is related to the similarities of the languages. For distant languages, a larger amount of storage is needed to handle two separate systems. In this case, the presence of a "control system" to prevent interference between the words of two distant languages is not necessary. On the other hand, when languages are similar, they have a shared system, which leads to the need for a control system to prevent interference between the languages. Similar to this view, Goral et al.^28^ and Kong et al.^29^ also suggest that two linguistic systems that are closely related may interfere and thus need increased control activity to keep the systems separate and to avoid potential interference during production. Likewise, Coderre and van Heuven^30^ suggested that similar-script bilinguals demonstrated more effective domain-general cognitive control than different-script bilinguals, since high orthographic overlap creates more cross-linguistic activation and increases the daily demands on cognitive control. This assertion was questioned by Paap et al.^13^ who stated that the effects of script similarity on bilingual advantages in cognitive control are likely to be negligible or null. Moreover, contrasting the view of stronger cognitive control involvement in close language pair bilinguals, Ghazi-Saidi and Ansaldo^31^ found that L2 picture-naming imposes a higher demand on cognitive control in phonologically distant language pairs (DLPs, Persian-French) than in close language pairs (CLPs, Spanish-French). This phenomenon was reflected in stronger recruitment of cognitive control areas (namely, the left inferior frontal gyrus, middle frontal gyrus, and bilateral cingulate gyri) during picture-naming in L2 in bilinguals of distant language pairs compared to bilinguals of close language pairs.

In summary, because of the limited data on the relation between linguistic distance and language control as well as discrepancies in the results, additional research is needed to understand the impact of L1–L2 relative distance on the language control system.

In the present study, two groups of healthy late bilinguals of a distant language pair (DLP, i.e., Persian-English) and a close language pair (CLP, i.e., French English) were recruited and asked to perform the following tasks while an electroencephalogram was recorded: (i) picture-naming in L1 and L2, (ii) picture-naming with L1–L2 switching and (iii) nonlinguistic switching.

The aim of the study was twofold. Aim 1 was to investigate to what degree language distance affects bilingual language control in L1 and L2 at the behavioral and electrophysiological levels. Based on the assumption that DLP bilinguals need to activate two separate lexica simultaneously^27^, we hypothesized that CLP bilinguals show shorter voice onset time (VOT) than DLP bilinguals in a linguistic switching task and that this effect would be stronger for switching into L1 than for switching into L2 in both groups (asymmetrical switching cost^32,33^). Alternatively, based on the assumption that the lexica of DLP bilinguals interferes less than those of CLP bilinguals^27,34^, one could also expect that CLP bilinguals require greater language control compared to DLP bilinguals and thus show longer reaction times in a language switching task. Actually, lexical decision tasks in order to measure costs in bilinguals when switching between languages suggest that language switch costs originate from a combination of both lexical access and a task-specific decision process^35^. The second process may depend on linguistic distances and may also depend on more general executive inhibitory processes. Until now, analysis pointed out that two different languages rely on their executive system, but also showed that groups of different distances (Swedish–Finnish/Swedish–English) did not show the influence of linguistic distance on switching behavior^36^. However, we re-evaluate this question with more distant languages. On the electrophysiological level, we expected differences between groups in time windows related to the inhibition of irrelevant information/cognitive control, namely, approximately 200 ms poststimulus onset. Moreover, we expected differential involvement of left lateralized frontal, prefrontal and subcortical structures^37^ between language switching and nonswitching conditions.

Aim 2 was to investigate whether language distance affects general cognitive control performance at the behavioral and electrophysiological levels. We expected that CLP bilinguals would show faster RTs than DLP bilinguals in a domain-general cognitive control task based on the speculation that there is greater interference between languages in CLP than DLP bilinguals, possibly leading to a cognitive control system that is more trained to high cognitive load, thus leading to faster RTs in a nonlinguistic switching task. On the electrophysiological level, it was thus expected that such a result would be reflected in differential involvement of cognitive control regions, including dorsolateral prefrontal and anterior cingulate cortices in time windows related to inhibition of irrelevant information/cognitive control^38,39^, i.e., approximately 200 ms.

## Materials and methods

Research methodology related to second language evaluation, task procedure and EEG data acquisition and analyses have been similar to our previous studies^40–42^.

### Participants

Twenty-nine native French speakers (n = 8 males, mean age 22.2 ± 3) were recruited for the CLP group, and 29 native Persian speakers (n = 14 males, mean age 27.1 ± 6) were recruited for the DLP group. All participants acquired English (L2) as a second language after 7 years of age. Subjects were right-handed according to the Edinburgh Handedness Inventory^43^ with no history of neurological or psychiatric illness or other health problems. Power analysis using G*Power^44,45^ indicated that a medium effect size (r > 0.4) can be detected with a power of 0.80 (alpha = 0.05 and medium effect size, dz > 0.60) with 25 participants per group^46^, in accord with the results of the previous studies.

The three languages spoken by the participants are part of Indo-European Language family. Persian is part of Indo-Iranian Language group. French is part of Italic group and English is part of the Germanic group. Lexical distance between French and English represents a direct link while distance between Persian and English represents an indirect link, corresponding categorically to a 3 steps distance^47^. Persian and English are different in orthography, morphology and phonology systems. Persian is not marked for gender and uses the Arabic writing system, whereas English uses the Latin alphabet. The canonical sentence order in English is Subject-Verb-Object (SVO), while in Persian, it is Subject-Object-Verb (SOV)^31^. English and French are considered closely related languages^48^. They share the same alphabet and a considerable amount of vocabulary and have closer morphology, orthography, and phonology.

### Second language evaluation

Participants completed the language experiences and proficiency questionnaire (LEAPQ^49^ to assess age of acquisition, immersion, and self-evaluation of language use. To assess L2 receptive vocabulary, the vocabulary subtest from the computer-based DIALANG language diagnosis system was administered^50^. In this test, participants indicated for each of the 75 presented stimuli whether they were English words or highly word-like pseudowords. This evaluation confirms an intermediate level of English vocabulary and no significant difference between the DLP and CLP DIALANG scores (independent sample t-test, t = 0.29, p = 0.77).

To assess L2 productive vocabulary, the PVLT (Productive Vocabulary Levels Test)^51^ was used. The PVLT samples 18 items at five different word-frequency levels; the first level representing the most frequent 2000 words and the four subsequent levels representing lower frequency words at several vocabulary frequency levels: 3000, 5000, University Word List (UWL) and 10,000. For simplicity, the four latter levels were merged into one score reflecting low-frequency words. According to Nation and Waring^52^, second language learners with knowledge of the most frequent 2000 words will know approximately 80% of the running words in a written or spoken text. Low-frequency words cover the remaining 20%. Table 1 provides details on participants’ L1 and L2-age of acquisition, proficiency skills and immersion.

**Table 1:** First and second language proficiency, usage and immersion.

Variable	DLP (L2)		CLP (L2)			DLP (L1)		CLP (L1)		
	Mean	SD	Mean	SD	p-value	Mean	SD	Mean	SD	p-value
**Self-evaluation**										
Speaking	7.28	1.95	5.75	1.69	0.74	9.03	1.68	9.57	0.69	0.003
Comprehension	8.14	2.47	6.78	1.25	0.12	9.64	0.82	9.75	0.51	0.19
Reading	8.46	1.13	6.57	1.39	0.37	9	1.92	9.71	0.59	0.08
**Exposure**										
Interacting with fiends	3.71	2.63	2.92	2.35	0.17	7.82	3.04	9.07	1.05	0.000
Interacting with family	1.85	2.53	0.53	1.23	0.003	7.85	3.25	9.64	0.98	0.000
Reading	7.64	2.72	4.42	1.81	0.13	6.92	2.74	7.57	1.66	0.02
Language-lab/self-instruction	5.60	2.58	1.03	1.73	0.07	3.64	3.62	0.71	2.17	0.000
Watching TV	6.03	3.45	5.10	2.73	0.13	5.75	3.69	6.60	2.58	0.002
Listening to radio/music	7.53	2.47	5.07	3.01	0.002	5.5	3.41	6.35	2.28	0.006
**Contribution to learning**										
Interacting with friends	4.57	3.09	5.46	3.01	0.87	8.5	2.64	8.57	1.64	0.043
Interacting with family	1.75	2.78	1.82	2.86	0.62	8.71	2.66	9.57	1.13	0.008
Reading	8.21	1.37	6.5	2.21	0.01	7.75	2.22	8.21	1.52	0.01
Language-lab/self-instruction	6.75	2.98	2.5	2.45	0.42	5.17	3.70	2.35	3.17	0.17
Watching TV	7	3.01	6.28	2.65	0.55	7.5	3.13	6.53	2.57	0.81
Listening to radio/music	4.78	3.66	2.10	2.78	0.07	5.64	3.76	5.03	2.31	0.001
English Exposure (%)	40.39	25.43	12.96	7.24	0.0001	57.5	26.38	71.75	9.46	0.000
**L2 vocabulary test**										
DIALANG score	712.57	165.53	723.40	99.41						
PVLT high frequency words	13.92	2.90	13.39	3.08328						
PVLT low frequency words	4.32	3.16	4.14	1.89						
PVLT total scores	45.28	15.18	43.21	9.36						
**Immersion**										
Use of at work/studies	3.45	5.26	0.35	0.66						
Order of Acquisition (N)										
	Persian (1)	28	French(1)	29						
	English (2)	27	English(2)	13						
	Azeri (2)	1	German (2)	15						

### Procedure, stimuli and task

The experiment took place at the Neuroscience Department of the University of Fribourg, Switzerland for the CLP group (French/English) and at the laboratory of the Institute for Cognitive Science Studies, Tehran, Iran for the DLP group (Persian/English). Participants first gave their written, informed consent and then completed the questionnaires and language proficiency tests. Participants’ written consent was obtained, and the study was approved by the Swiss and Iranian local ethics committees (Vaud cantonal human research ethics committee, Switzerland, ref. no: 2019-00939, and SCS research ethics committee, ref. no: 98/60.1/1661, respectively). The first author was present in both experimental places in at least part of the recordings to ensure the similarity of procedures across countries. The experiment is done in accordance with Declaration of Helsinki (BMJ 1991; 302: 1194).

During the 64-electrode EEG acquisition, participants sat comfortably in a sound attenuated room at 110 cm from a 19′ LCD screen on which the stimuli were presented. Participants were asked to perform the following tasks while the EEG was recorded: 1. The picture-naming task, consisting of (a) language nonswitching and (b) the language switching condition and 2. a nonlinguistic switching task. The procedure of these tasks have been adapted from our previous studies^41,42^.

#### Picture-naming tasks

##### Nonswitching picture-naming

Stimuli consisted of one list of 70 images to be named in French and English by the CLP bilingual participants and to be named in Persian and English by the DLP bilingual participants. Items were selected from the Snodgrass image corpus^53^. Target names were noncognates, and word frequency, name agreement, and image agreement were matched across the three languages based on data reported by Alario and Ferrand^54^ and Bakhtiar, Nilipour and Weekes^55^. Persian words were phonologically longer than English and French words (*F*(2, 67) = 7.18, *p* = 0.001, followed by a post hoc Tukey test). All images consisted of line drawings of approximately the same size (no larger than 540 × 400 pixels) with a white background. Participants were asked to name pictures as quickly as possible. Stimuli were presented in a random order for 2000 ms either in the upper or lower part of the screen with a refresh rate of 60 Hz. Each picture was preceded by a fixation cross of a random duration of 500–1500 ms. A fixation cross of 1000 ms followed the picture.

This task was performed in L1 and L2 in separate blocks for each group.

##### The language switching picture-naming task

The stimuli for this task consisted of the same list of 70 images, which was used for the nonswitching picture-naming task. Each image was presented twice, once it had to be named in L1 and once in L2. Therefore, this task consisted of a total of 140 trials. A similar task procedure was applied. The subjects were asked to name the images in L1 when the image appeared in the upper part of the screen and name the image in L2 (English) when it appeared in the lower part of the screen. The order of the type of trials was kept constantly alternating. This method of stimuli presentation resulted in 70 L1-switches (i.e., a switch into L1) and 70 L2-switches (i.e., a switch into L2) trials. The order of image presentation was random.

In the picture-naming tasks (language switching and nonswitching), the oral responses were recorded in 3-s files for each trial. Only first-attempt correct responses (i.e., the exact expected word or close alternative responses as listed in the image corpus normative data for each language) within three seconds of the presentation of the image were scored as correct. In addition, the beginning of oral production for each trial was captured (i.e., voice onset time, VOT).

#### Nonlinguistic switching task

Adapted from our previous work^41^ and based on a language execution paradigm^56^, this task consisted of 140 trials of four types of images (a red or blue circle or square). At each trial, one image was presented on the upper or the lower part of the screen. Subjects were instructed to press the corresponding response key as quickly as possible, to select the color of the image when the image was presented in the upper part of the screen and to select the shape of the image when it was presented in the lower part of the screen. Four keys on the 5-botton Chronos box were assigned to 4 response types (square, circle, red and blue). Participants were instructed to use both hands and their index and middle finger to allow fast responses. Two versions of the task were designed and counterbalanced across participants.

After a fixation cross of 500–1500 ms, the images were presented on the screen for 2000 ms and were followed by a fixation cross of 1000 ms. The order of stimuli presentation was alternating shape-color.

At the beginning of the session, a short training was performed for each task to familiarize participants with the procedure. For the picture-naming tasks, the training consisted of five trials with stimuli that were not part of the main experiment.

**Figure 1:**
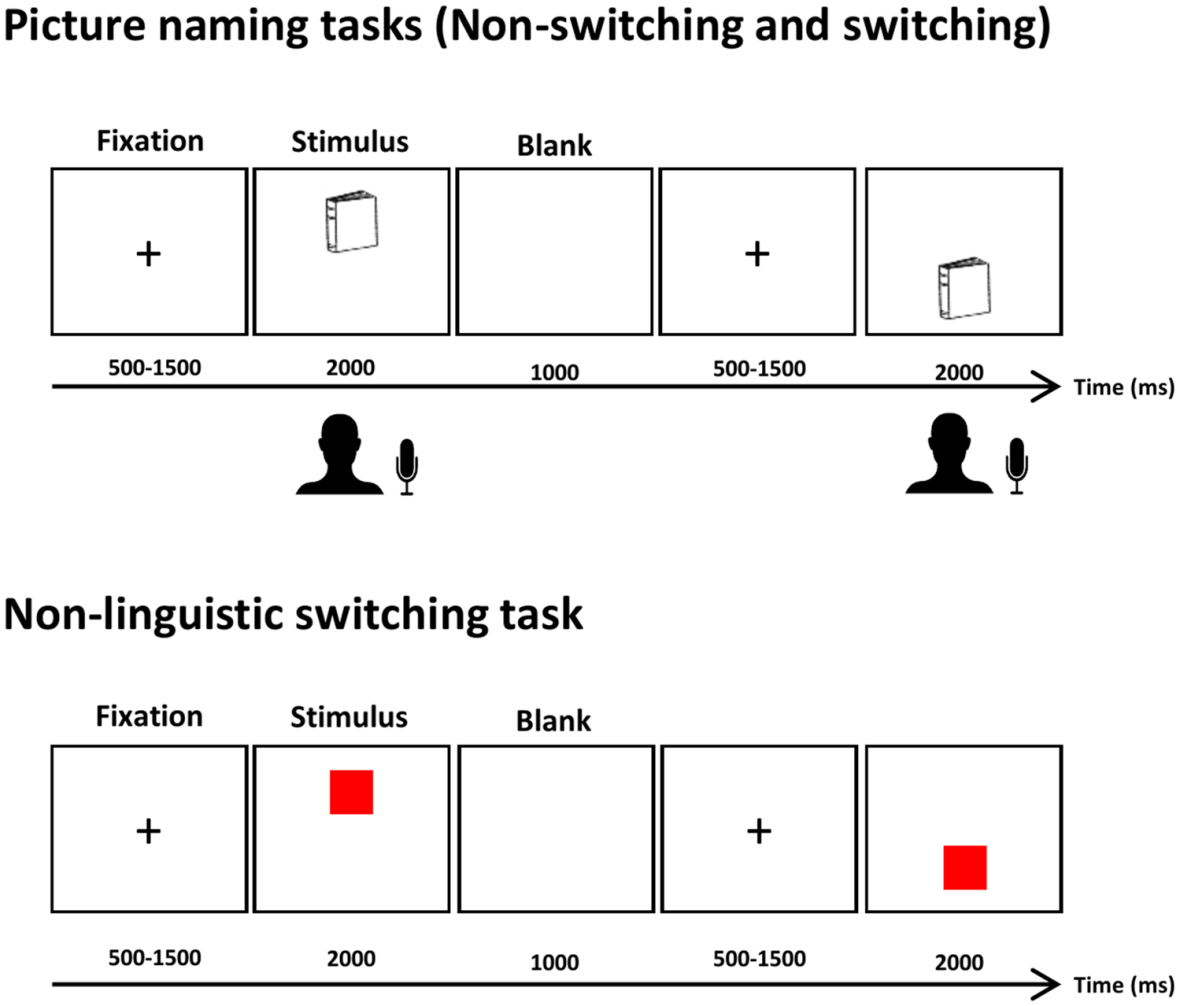
Task procedure; switching and nonswitching picture-naming and nonlinguistic switching tasks. In switching picture-naming (i.e., the linguistic switching task) participants had to name the pictures in L1 when the picture appeared on the top and name the picture in L2 when it appeared on the bottom of the screen.

Stimulus presentation and behavioral data recording were controlled with EPrime 2.0 (Psychology Software Tools, Inc., Sharpsburg, PA, USA). Figure 1 illustrates the procedure of the tasks. The task order was counterbalanced across participants.

Both behavioral and EEG data that support the findings of this study are available from the corresponding author upon reasonable request.

### Analysis of behavioral data

Each spoken response was checked for accuracy and the VOT. For this work we used version 2.4.2 of AUDACITY(R) recording and editing software^57^. This process allows for adjustments of the VOT in case of hesitations, audio artifacts and autocorrections.

Behavioral data analyses were conducted using mixed-effects models using R^58^ and *lme4*^59^, to find the model formula which best fit the data, in order to investigate aim1, i.e., to what degree language distance affects bilingual language control in L1 and L2. As fixed effects, we entered Response Accuracy, Language, Switching and Group (with interaction terms) into the model. As random effects, we had intercepts for Subjects, L2 exposure and Items, as well as by-subject, by-L2 exposure and by-item random slopes for the effect of Accuracy, Language, Group and Switching. Visual inspection of residual plots did not reveal any obvious deviations from homoscedasticity or normality. P-values were obtained by likelihood ratio tests of the full model with the effect in question against the model without the effect in question. As the reference levels we took CLP Group, Accuracy 0 (incorrect responses), Non-Switch and L1.

We used R^58^ and *lme4*^59^ to perform a linear mixed effects analysis of the relationship between response time (RT) of nonlinguistic switching task and language distance and finding the model which best fits the data. As fixed effects, we entered Response Accuracy and Group (with no interaction term; F(42.76) = 1.25, p = 0.27) into the model. As random effects, we had intercepts for Subjects and Items, as well as by-subject and by-item random slopes for the effect of Accuracy. Visual inspection of residual plots did not reveal any obvious deviations from homoscedasticity or normality. P-values were obtained by likelihood ratio tests of the full model with the effect in question against the model without the effect in question. As the reference levels we took CLP Group and Accuracy 0 (incorrect responses). To decrease the complexity in reporting the behavioral results, we report significant results only.

### EEG acquisition and preprocessing

Electroencephalograms were recorded continuously at 1024 Hz from 64 Ag/AgCl electrodes using an ActiveTwo system (BIOSEMI, Inc., Amsterdam, the Netherlands) for CLP bilinguals in Switzerland and an ANT Neuro with Waveguard caps (ANT NEURO, the Netherlands, www.ant-neuro.com) for DLP bilinguals in Iran. Electrodes were placed in an elastic cap according to the 10–20 system, the reference electrode was placed at the vertex (“Cz”), and impedances were kept below 5 kΩ for the ANT Neuro system for the DLP group. For EEG recording of the CLP group with ActiveTwo, electrode-offset values (i.e., running average of voltage at each electrode relative to the common mode voltage of the system) of ± 30 mV were used as a quality check of the EEG signal. Offline, the data were recomputed to the average reference and digitally bandpass filtered between 0.1 and 40 Hz using a second-order Butterworth filter with a – 12 db/octave roll-off. Additionally, a notch filter (50 Hz) was used to remove the alternating current (AC) noise.

To obtain comparable montages in both systems, we performed the analyses only on the channels common to both EEG systems, which resulted in a 60-channel montage (Fig. 3A). All offline analyses were performed using CARTOOL software by Denis Brunet and the STEN toolbox developed by Jean-François Knebel and RAGU software developed by Thomas Koenig^60^. To avoid possible topographic distortions caused by electrode bridges, the eBridge toolbox in EEGLAB was used to find any possible bridges in the raw EEG files^61^.

For each trial, epochs from 100 ms prior and 600 ms post presentation of the stimuli were extracted. The extracted epochs of each participant were averaged to calculate the event-related potentials (ERPs) for each condition of interest separately, that is, (a) picture-naming in L1, (b) picture-naming in L2, (c) picture-naming switching between L1 and L2, and (d) nonlinguistic switching. Only trials with correct responses were included. To avoid artifacts related to overt speech production, trials with VOTs shorter than 600 ms were removed for the linguistic tasks. Epochs with eyeblinks or other artifacts (i.e., amplitude changes exceeding 80 μV on at least one electrode during an epoch) were rejected before averaging^62^. Electrodes exhibiting substantial artifacts as well as bridged electrodes were interpolated (on average, 5.1% of electrodes were interpolated) using a three-dimensional spline algorithm^63^. Only participants whose accepted trial number was above 20 epochs in each of the conditions were included in the analysis. As such, data from 21 DLP participants and data from 20 CLP participants were included for the analyses of the picture-naming tasks. For the nonlinguistic switching task, 20 participants per group were included.

The average number of accepted epochs was 44.1 ± 13.2 for the picture-naming tasks and 103.1 ± 28.5 for the nonlinguistic switching task. The number of accepted epochs did not differ significantly between conditions of interest. This procedure ensures that the observed ERP effects were not confounded by differences in the signal-to-noise ratio.

### Electrical neuroimaging data analysis

The EEG analyses were similar to the analysis approach in our previous studies (For more details on the analysis methods see Refs.^40,42^). We used global analyses for the analysis of the ERPs, namely, analyses of global field power (GFP) and topography^40,41,64^. Such analyses are reference-independent^65^ and allow verification of whether the observed effects are caused by differences in activation strength or are due to differences in the configuration of the underlying active brain generators across experimental conditions (e.g., Refs.^66–68^).

#### Global field power analysis

The electric field strength differences at the scalp were examined by computing a nonparametric randomization test on the GFP^60,69,70^. The GFP equals the root mean square across all recording electrodes and represents the spatial standard deviation of the electric field at the scalp^70^.

#### Global dissimilarity analysis

Topographic modulations were analyzed using randomization statistics applied to global map dissimilarity measures (GMD^70^). GMD is calculated as the root mean square of the difference between the strength-normalized voltage potentials across the electrode montage. Applying this analysis to the strength-normalized data allows attributing topographic differences between conditions to differences in source distribution and not in source strength (e.g., Ref.^71^). GMD values were analyzed as a function of peristimulus time with respect to the ERP analyses^60,69,72^. A total of 5000 randomization runs were computed on subject-level averaged epochs (for details see Refs.^60,69^, with the p-threshold set to 0.05). Correction was made for temporal autocorrelation through the application of > 11 contiguous data points temporal criterion for the persistence of significant effects^73^.

For the ERPs of the picture-naming tasks, both the GFP and the GMD were analyzed by applying a three-way ANOVA with the within-subject factors Language (L1; L2) and Switching (Switch; Non-Switch) and the between-subjects factor Group (CLP; DLP). To answer the specific question concerning the role of linguistic distance on language selection in both languages, the three-way interaction was examined.

Regarding the ERPs of the nonlinguistic switching task, given that the task was not language related and that there were only switching trials, the only effect of interest was the difference between the two groups. Therefore, differences in GFP and GDM between the CLP and DLP groups were analyzed using independent samples t-tests.

#### Analysis of intracranial sources and source differences

To estimate intracranial generators, we used a distributed linear inverse solution based on the local autoregressive average (LAURA) regularization approach^65,74^. LAURA selects the source configuration that best mimics the biophysical behavior of the electric fields; it confines the solution space in the gray matter of the brain and considers how the activity in a given area reduces with the distance from the scalp and thereby assumes smoothness between adjacent sources. The solution space is based on a simplified realistic head model (SMAC^75^) and contains 5006 solution points homogeneously distributed within the gray matter of the average brain of the Montreal Neurological Institute (MNI). This was used as the template source space for all subjects.

The lead field (or the forward solution) was then solved with an analytical solution with a three-shell spherical head model (brain, skull, and scalp)^76,77^.

The intracranial sources were estimated for the resulting one time-sample ERP for each subject, and each condition was time-averaged over the period of interest (i.e., the period showing a significant topographic interaction or difference) and then statistically compared using the same mixed within-between-subject design for picture-naming tasks and group comparison for the nonlinguistic switching task as was used for the GDM analysis. To control for multiple comparisons, only significant clusters of > 15 consecutive points (*K*E) were kept. This spatial criterion was determined with the AlphaSim program. There was a false positive probability of *p* < 0.005 for observing a cluster of > 15 contiguous nodes (see also Refs.^78,79^ for the same approach). For the resulting regions of interest (ROIs), we extracted and analyzed the T values, which then allowed us to draw conclusions on the direction of the effects, namely, whether the solution points found are more or less activated in the conditions of interest.

## Results

### Behavioral results

#### Picture naming task

Including L2 exposure as a random factor led to singularity problems and was removed from the model. The mixed-effects linear model on VOT [intercept: estimate = 1277.41 (SE = 63.36, t = 20.162, p < 0.001)] revealed a main effect of Accuracy [estimate = − 349.09 (60.63), t = 9.50, p < 0.001, shorter VOTs in correct responses], Switching [estimate = 101.97(39.62), t = 2.574, p = 0.01, longer VOTs in Switch trials], and Language [estimate = 249.21 (SE 44.43), t = 5.609, p < 0.001, longer VOTs in response to L2 trials].The model showed interaction between Accuracy, Group, Switching and Language [estimate = 395.27 (84.98), t = 4.652, p < 0.001], led by longer VOTs of correct responses to Switch to L2 compared to Non-Switch trials in the DLP group compared to the CLP group. The details of these results can be found in the Supplementary Information.

The model formula which best fit the data is the following:

$$VOT \, \sim \, Accuracy \, \times \, Group \, \times \, Switching \, \times \, Language \, + \, \left( {Accuracy + \, Language \, |Item} \right) \, + \, \left( {Accuracy + \, Language \, |Subject} \right).$$

#### Nonlinguistic switching task.

The results for our model [estimate = 903.53 (SE 65.60, t = 13.774, p < 0.001] showed that Group [estimate = 134.99 (SE 58.11), t = 2.323, p = 0.0242] but not Accuracy [estimate = − 26.62 (SE 58.89), t = − 0.452, p = 0.6552] were linearly related to RT. This revealed longer RTs to nonlinguistic switching trials in the DLP group. The details of these results can be found in the supplementary file.

The model formula which best fit the data was:

$$RT \, \sim \, Accuracy \, + \, Group \, + \, \left( {Accuracy \, | \, Subject} \right) \, + \, \left( {Accuracy \, | \, Item} \right).$$

**Figure 2:**
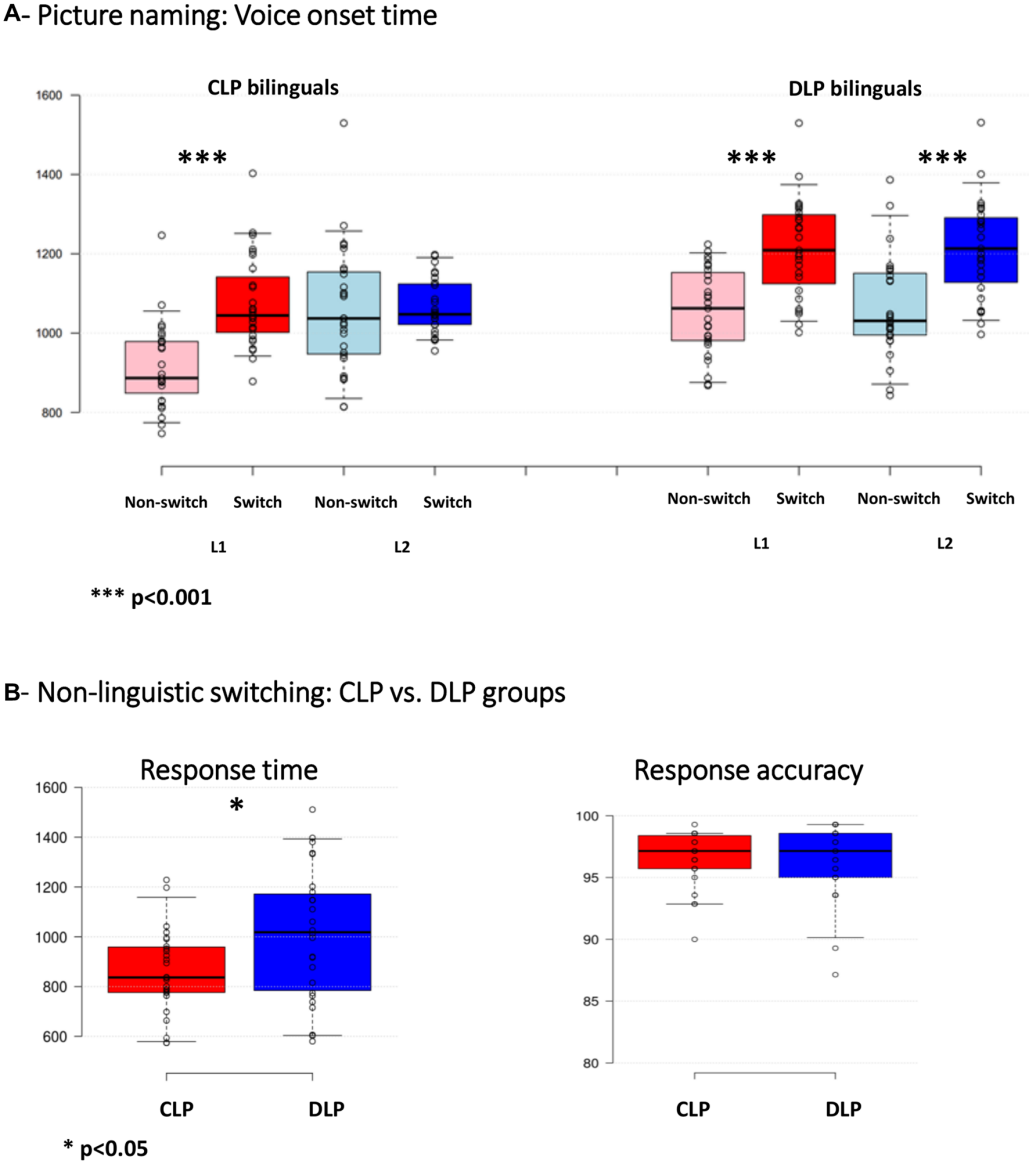
Behavioral results; (**A**) Picture-naming voice onset time. (**B**) Nonlinguistic switching task, response accuracy and response time.

Figure 2A,B illustrate behavioral results.

Given that approximately one-third of the participants in each group had to be removed from the EEG analyses (see section “EEG acquisition and preprocessing”), we also performed the same behavioral analyses on the specific subgroup of participants included in the EEG analyses. The behavioral analyses on this subgroup revealed a similar pattern of results as was the case for the initially included participants. Therefore, we report the behavioral analyses of the initial groups.

### Electrical neuroimaging results

#### Picture-naming tasks

**Figure 3:**
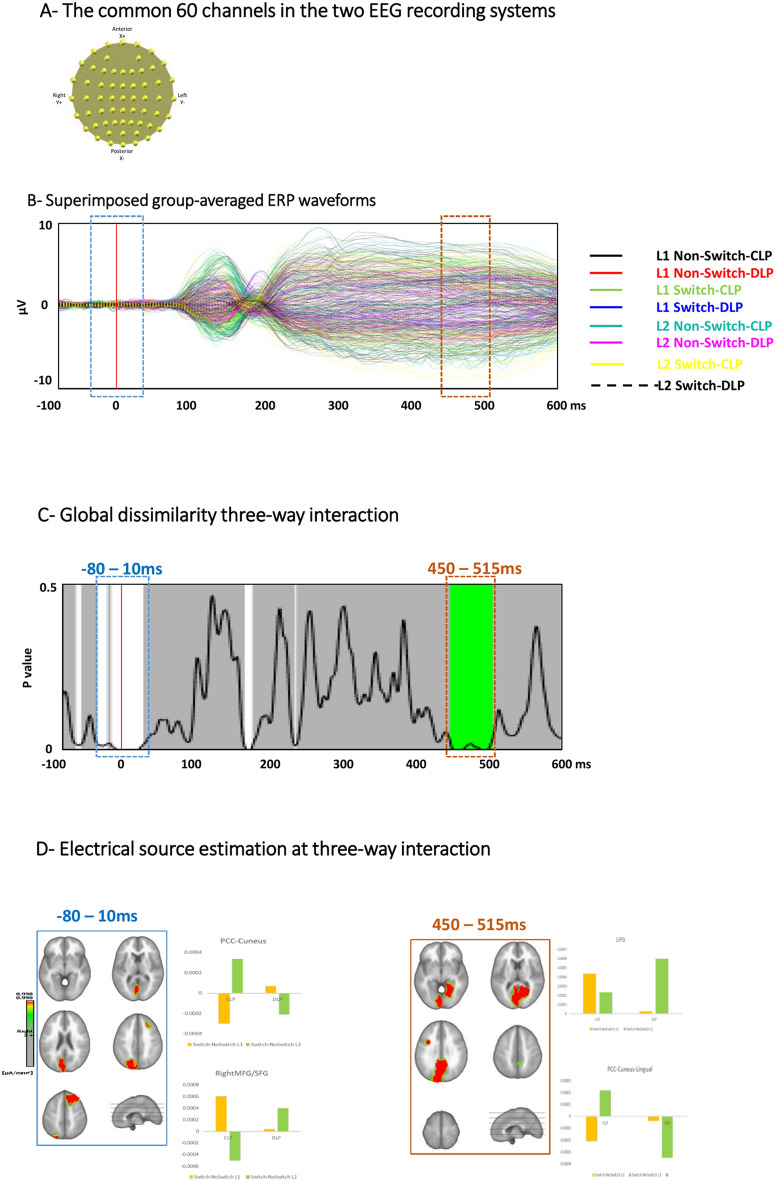
Electrical neuroimaging results: The picture-naming task; (**A**) The 60 common electrodes in the two EEG systems. Although both EEG recording systems consisted of 64 electrodes, only 60 of them had similar coordinates. These 60 channels were kept for the analyses. (**B**) Superimposed group-averaged ERP waveform of all conditions of interest. (**C**) Global dissimilarity analysis [Language (L1; L2) × Switch (Non-Switch; Switch) × Group (CLP; DLP)]. This analysis showed two time windows of significant interaction at − 80 to 10 ms peristimulus and 450 to 515 ms poststimulus presentation. (**D**) Electrical source estimation at the time windows of the significant three-way interaction.

All conditions (L1 Non-Switch naming, L1 Switch naming, L2 Non-Switch naming, and L2 Switch naming) resulted in event-related potential waveforms that are typical for visual stimuli, as shown by superimposed group-averaged ERPs in Fig. 3B.

##### Global Field Power (GFP) analysis on picture-naming tasks

In the GFP analysis, no significant periods of a three-way interaction were found (all p > 0.05). However, a significant Language × Switching interaction was found from 410 to 500 ms poststimulus onset (p < 0.05, > 46 TF), while the two-way interactions between Switching × Group and Language × Group did not reach significance (both p > 0.05). Moreover, there was a main effect of language showing higher GFP for L2 compared to L1 naming from 500 to 600 ms poststimulus onset (p < 0.05, > 46 TF). The analysis on the GFP also showed a main effect of Switch, revealing higher GFP for Non-Switch than Switch conditions from − 100 to 90 ms and from 310 to 470 ms (p < 0.05, > 46 TF). The time windows found for the main effects and Language × Switching interaction occurred at the same time as topographic differences, which indicates differences in underlying neural sources (see below). GFP differences in the presence of topographic differences could mainly be explained by changes in topography (i.e. changes in underlying active sources) and not change of the activation strength of specific brain regions. As such, this result indicates that the differences are of a topographic nature rather than based on differences in GFP.

##### Global map dissimilarity (GMD) on picture-naming tasks

The topographic analysis revealed a three-way Language × Switching × Group interaction (p < 0.05, > 50 TF) from − 80 to 10 ms and from 450 to 515 ms, indicating that the topographic maps were significantly different in the CLP and DLP groups depending on the Language (L1 or L2) and Switching condition (Switch or Non-switch context) (Fig. 3C).

The topographic analyses revealed no significant two-way interactions for the factors Switching × Group or Group × Language (all p > 0.05). However, a Language × Switching interaction as well as long-lasting main effects of Language and Switching were found during the entire peristimulus time window. The main effect of Group was not significant.

Additional global duration statistics revealed that 50 consecutive time frames can be expected at the chance level of 0.05, which was the case for the time windows revealing a three-way interaction and the main effects of Language and Switching.

##### Electrical source estimation on picture-naming tasks

Considering the main aim of the study, source localization was performed on the period of the three-way interaction. Repeated measures ANOVA of distributed source estimates for all conditions was performed for each of the 5006 solution points for the period of significant effects identified in the GMD analyses, i.e., from − 80 to 10 ms peristimulus and 450–515 ms poststimulus presentation for the picture-naming tasks.

The significant topographic Language × Switching × Group interaction in the peristimulus period of − 80 to 10 ms was driven by the stronger activation for the L1 Switch and L2 Non-Switch in the right MFG/SFG of the CLP group. In the DLP group, higher activation in the right MFG/SFG was revealed in L2 Switch, but no difference between Switch and Non-Switch was seen for L1 naming. A reverse pattern was seen in the posterior cingulate cortex (PCC), cuneus and lingual gyrus (LG) with stronger activation in L1 Non-Switch and L2 Switch of the CLP group, while higher activation of these posterior regions was observed in response to L2 Non-Switch in the DLP group.

Topographic differences in the time period of 450–515 ms were characterized by a higher activation in the left inferior frontal gyrus (IFG) for L2 Switch compared to L1 Switch in the DLP group, while in the CLP group, both L1 and L2 Switch naming showed strong activation in the left IFG (higher activation for L1 Switch). In the posterior regions, a similar pattern of activation as was the case in the peristimulus time range was observed. (Fig. 3D).

#### The nonlinguistic switching task

##### Global Field Power (GFP) analyses on nonlinguistic switching tasks

The GFP analysis revealed a period of differences between the CLP and DLP groups from 110 to 135 ms poststimulus onset, with higher GFP for the CLP group than for the DLP group. However, this effect did not pass the test of multiple comparisons according to which at least 48 consecutive significant time frames can be expected at a chance level of (p < 0.05, < 48 TF).

##### Global map dissimilarity (GMD) on nonlinguistic switching tasks

**Figure 4:**
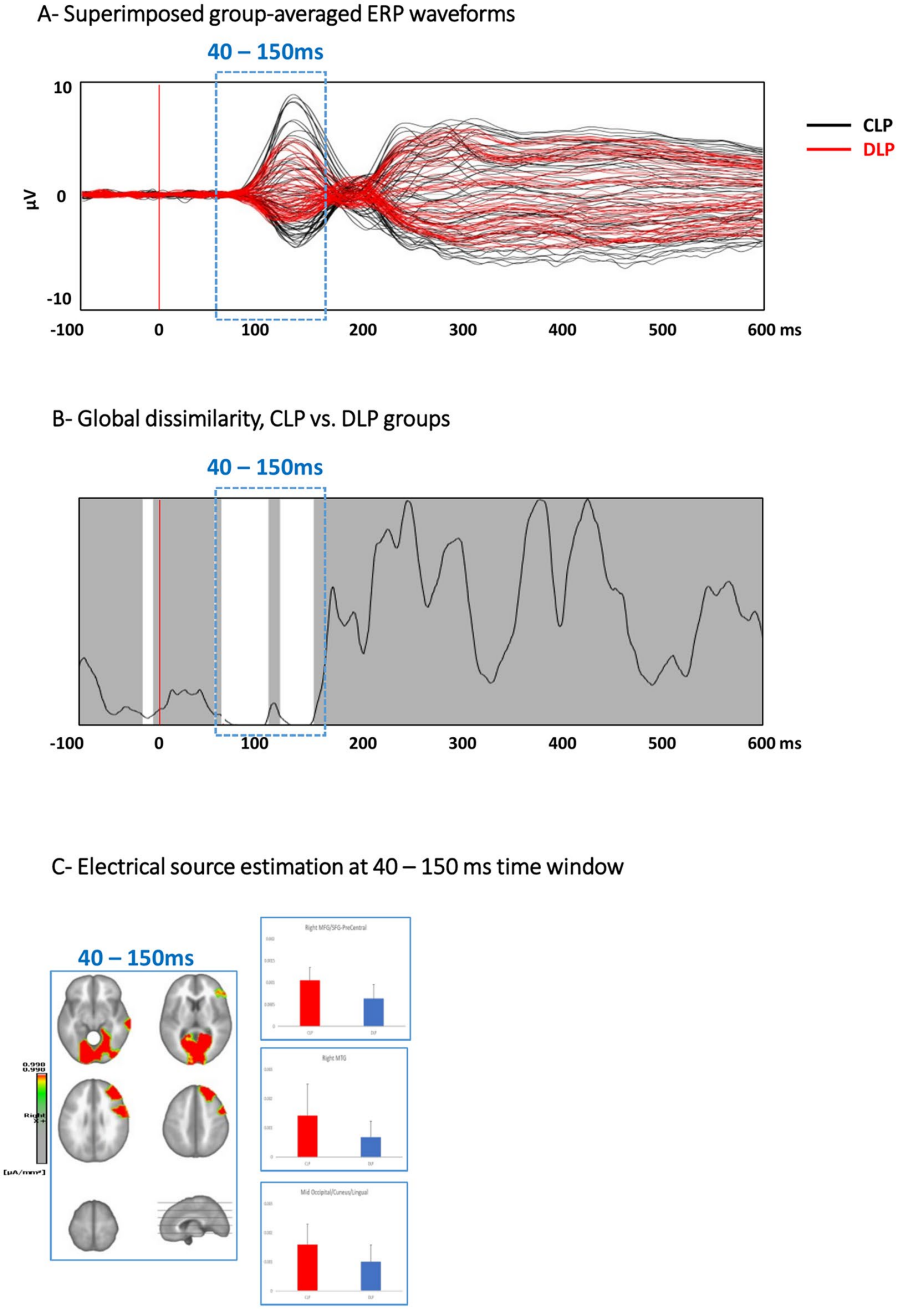
Electrical neuroimaging results: Nonlinguistic switching task; (**A**) Superimposed group-averaged ERP waveform of all conditions of interest. (**B**) Global dissimilarity analysis. This analysis showed a significant difference between CLP and DLP at 40 to 150 ms poststimulus presentation. (**C**) Electrical source estimation at the significant time windows.

The topographic analysis revealed a significant difference between the CLP and DLP groups from 40 to 150 ms poststimulus presentation (p < 0.05, > 96 TF) (Fig. 4A shows superimposed event-related potentials to different conditions and Fig. 4B illustrates global map dissimilarity results).

##### Electrical source estimation on nonlinguistic switching tasks

The analyses of the source estimates for all conditions were performed for each of the 5006 solution points for the period of significant effects identified in the GMD analyses, i.e., the picture-naming tasks and from 40 to 150 ms for the nonlinguistic switching task.

The topographic differences between the CLP and DLP groups in the nonlinguistic switching task in the time period of 40–150 ms were characterized by stronger activation for the CLP compared to the DLP group in the right middle/superior frontal gyrus (MFG/SFG) and precentral gyrus (PrG) (Fig. 4C).

### Summary of the results

In the picture-naming task, linear mixed effect modelling analyses of the VOTs revealed longer VOTs of correct responses to Switch to L2 compared to Non-Switch trials in the DLP group compared to the CLP group (i.e. Accuracy × Group × Switching × Language interaction (estimate = 395.27 (84.98), t = 4.652, p < 0.001). The VOTs was not significantly affected by L2 exposure.

Electrophysiologically, in topographic analyses, we found Language × Switching × Group interaction at − 80 to 10 ms and 450 to 515 ms peristimulus presentation. The prestimulus effect was observed in the right MFG/SFG. The significant interaction at 450–515 ms post stimulus involved the left IFG as well as the posterior cingulate cortex (PCC) and occipital regions.

In nonlinguistic switching task, Group, but not Accuracy, was linearly related to RT. This revealed longer RTs to nonlinguistic switching trials in the DLP group.

Electrophysiologically, our topographic analysis revealed a significant difference between the CLP and DLP groups from 40 to 150 ms poststimulus presentation. This topographic difference was characterized by stronger activation for the CLP compared to the DLP group in the right middle/superior frontal gyrus (MFG/SFG) and precentral gyrus (PrG).

## Discussion

The present study investigated the role of linguistic distance on cognitive control using linguistic- and nonlinguistic switching tasks. Two groups of bilinguals, namely, Close Language and Distant Language Pair bilinguals, were recruited (i.e., CLP and DLP groups). Language-switching, language-nonswitching and a nonlinguistic-switching tasks were performed.

### Language switching vs. nonswitching picture-naming

#### Behavioral data: switching cost

In the picture-naming task, linear mixed effect modelling analyses of the VOTs revealed longer VOTs of responses to Switch to L2 compared to Non-Switch trials in the DLP group compared to the CLP group. This result can be explained according to the assumption that DLP bilinguals involve more language control in a bilingual context^31,80^, and as a result, they show slower language switching compared to nonswitching. Interestingly, a different pattern of VOTs in response to Switch vs. Non-Switch naming was found for L2 vs. L1 in the CLP group; while the DLP group showed slower VOTs for Switch trials into L1 and L2, CLP bilinguals showed slower VOTs for Switch trials into L1 but similar VOTs for Non-Switch and Switch trials into L2.

Such an asymmetric switching cost, showing longer VOTs for switching into the more dominant L1 than into L2, has previously been observed in language switching paradigms^30,33,81^ and has first been described by Meuter and Allport^82^, as “paradoxical” asymmetry in the language switching cost which is well explained by the inhibitory control model (IC) of Green^83^. According to the IC model, in bilinguals, when speaking in the more dominant L1, not much inhibition is required to suppress the less dominant L2, especially in less proficient bilinguals. However, when speaking in L2, the inhibition of L1 is more demanding. In a language switching context, this inhibition of L1 persists into the following trial. Thus, when switching back to L1, more time is needed to overcome the inhibition of the previous trial, leading to longer VOTs when switching to L1 but not to the less-dominant L2. Importantly, this outcome was not observed in DLP bilinguals; these participants showed a similar switching cost for L1 and L2. This asymmetry has been mostly described in receptive language tasks but rarely in naming tasks^84^. The differential pattern of switching cost between CLP and DLP bilinguals could be explained in terms of language distance. That is, in DLP bilinguals, because less competition occurs between L1 and L2 representations^27,28^, the inhibition of L1 and L2 is relatively similarly demanding. Therefore, we may assume that the difference of L1 and L2 inhibition was not present in DLP group, and paradoxical asymmetry in switching cost was not observed in DLP bilinguals. Alternatively, this differential pattern of switching cost in CLP and DLP bilinguals somehow confirms the higher efficiency of cognitive control in CLP groups. Because CLP bilinguals need a stronger involvement of the control system to prevent interference between the languages^27,28,85^, their cognitive control system is “more trained” for switching conditions and thus can handle switching to L2, which needs inhibition of the dominant L1, more efficiently. Studying a case of a trilingual Hebrew-English-French aphasic patient, Goral et al.^28^ suggested that multilingual lexical access can be affected by the relative degree of shared language systems. Coderre and van Heuven^30^ tested three groups of bilinguals with language pairs of high to low script similarity (German-English, Polish-English and Arabic-English, respectively) and a group of English monolingual individuals on a Stroop and Simon task. Arabic-English bilinguals showed the smallest Stroop interference effects but the largest overall RT in both tasks. The authors suggested that because high orthographic overlap creates more cross-linguistic activation and increases the daily demands on cognitive control, similar-script bilinguals demonstrate more efficient domain-general executive control than different-script bilinguals.

Importantly, adding L2 exposure to the linear mixed model caused singularity problems (i.e. random effect variance estimates of (nearly) zero). This led to the assumption that the VOTs (our independent variable) were not significantly affected by L2 exposure. It is also noteworthy that because the order of the task was counterbalanced and the stimuli were presented in a random order, the order effect was similar across both groups, suggesting that there was no order bias across interactions.

#### Electrophysiological data: process of language selection

Electrophysiologically, our results showed a Language × Switching × Group interaction at − 80 to 10 ms and 450 to 515 ms peristimulus presentation. These effects were related to differences in activation of the underlying sources. The three-way interaction observed in the prestimulus time range (− 80 to 10 ms) is most likely related to preparatory processes and voluntary reorientation of attention, indicating possible differences in brain activity before stimulus presentation, as the trials were presented in separate block (in non-switch naming) or predictable language order (in the language-switching task)^40,86^. Importantly, this prestimulus effect was observed in the right MFG/SFG. In previous studies, the right MFG has been considered a hub for proactive filtering and suppressing distracting information^87^. Moreover, a role in task switching has also been reported^88^.

The significant interaction at approximately 450–515 ms post stimulus involved the left IFG as well as the posterior cingulate cortex (PCC) and occipital regions. In non-switching picture- naming paradigms, this time window, according to Indefrey and Levelt (2004), is related to the processes involved in form encoding (accessing phonological code, syllabification and phonetic coding). Based on a large meta-analysis, Indefrey and Levelt^89^ suggest that word production starts by accessing the lexical concept from a visual object approximately 175 ms after picture presentation, followed by lexical selection/lemma retrieval at 150–350 ms. Accessing the phonological code, syllabification and phonetic encoding take place between 350 and 600 ms, followed by the articulation of the word. However, according to the IC model^83^, because in bilingual language production, both target and nontarget languages are coactivated and compete to be selected, an additional process in bilingual language production is language selection. In the same way, Christoffels et al.^90^ performed an ERP study on bilingual language switching in a picture-naming study. In this study, the authors found an ERP deflection between 375 and 475 ms poststimulus presentation. The authors reported main effects of Switching context (increased negativity in response to non-switch naming), Language (a more negative amplitude in response to L1 than L2), and cognate status (higher amplitude for cognates). The authors suggest that this ERP modulation represents the switching costs and monitoring lexical conflict. According to the authors, this pattern of results may distinguish the “default” speech condition (L1 naming in the nonswitch condition) from the other conditions. Our results support the findings of Christoffels. Thus, we could assume that this effect happens during the process of language selection. This interpretation is supported by the analyses performed in the brain space over the same time range (450–515 ms), showing a differential pattern of activation in the left IFG and PCC for L1 and L2 Switch in the CLP and DLP groups. The left IFG is shown to be involved in suppressing prepotent but incorrect responses^91^. In bilinguals, the left IFG has been shown to be involved in language control during switching naming tasks^92^ and domain-general cognitive control^93^. The PCC is a central part of the default mode network (DMN), which shows a baseline activity and is rapidly deactivated during attentionally demanding tasks (see Ref.^94^ for a review). In addition, the PCC is reported to play a crucial role in internally directed cognition, arousal and awareness, episodic memory retrieval and regulating the focus of attention specifically in high-attentional demanding situations (e.g., Refs.^40,94,95^). Interestingly, the differential pattern of activation in the left IFG and PCC for L1 and L2 Switch and Non-Switch conditions were inversed in the present study; the left IFG showed a higher activation in response to L2 Switch in the DLP group and both L1 and L2 Switch in the CLP group. In contrast, the PCC is less activated in response to L2 Switch in the DLP group and L1 Switch in the CLP group, the two conditions that are considered to impose more cognitive demand and higher arousal.

Together, our behavioral and electrophysiological results suggest that CLP bilinguals show stronger involvement of the control system when performing a language-switching task. We may note that the observed pattern of neural recruitment can be explained as the effect of adaptation to potentially stronger cognitive control demands due to more overlap across aspects of the two languages. This neural recruitment is more prominent for switching into L1. This stronger involvement of the control system leads to a more efficient switching performance in CLP bilinguals than in DLP bilinguals.

### Nonlinguistic switching performance in CLP and DLP bilinguals

CLP participants showed faster responses in the nonlinguistic switching task than DLP participants. Electrophysiologically, we found different activation of the underlying sources between the CLP and DLP groups from 40 to 150 ms with stronger activation for the CLP compared to the DLP group in the right middle/superior frontal gyrus.

The observed difference between CLP and DLP bilinguals in nonlinguistic switching performance confirms our primary hypothesis of faster RTs in CLP than in DLP bilinguals. This result is in line with the results of Coderre and van Heuven^30^, suggesting that bilinguals of similar languages, with similar scripts, experience more effective executive control than different-script bilinguals. In contrast, this pattern of results does not support our hypothesis, according to which we would expect faster RTs in DLP bilinguals, which is due to the less-trained cognitive control system in CLP bilinguals because of the possible facilitatory effect of similar lexica. In the same way, our results are not in line with Oschwald et al.^80^. According to Oschwald and colleagues, who studied three groups of bilinguals with different linguistic similarities and a group of monolingual individuals, language similarity is linearly related to linguistic accuracy. Oschwald et al. postulate an inverse pattern for performance in executive function: better EF performance with more dissimilar languages. In children learning L2, Floccia et al.^96^ found better working memory scores if L2 was distant from L1 but ambiguous results for inhibition.

The early effects from 40 to 150 ms showing stronger right frontal activation in CLP than DLP bilinguals are most likely associated with attentional processing. Given that the order of stimuli presentation was alternating between shape-color, the type of the following stimulus was predictable to the participants, thus potentially triggering voluntary reorientation of attention and response preparation^97^. This interpretation is further supported by the pre-stimulus differences, which did not last long enough to pass the temporal criterion for the persistence of significant effects, but which were nevertheless detectable. Such pre-stimulus differences have been found in other ERP studies, especially those employing blocked designs, therefore increasing predictability of the upcoming stimulus^97^. Our result thus indicates that the CLP and DLP bilinguals differ in regard to the recruitment of preparatory neural resources.

The estimated source localization revealed stronger activation for the CLP compared to the DLP group in the right (medial and superior) frontal regions, right middle temporal gyrus and the PCC. Previous studies found involvement of the right frontal regions in the inhibition of behavioral responses in task switching. More specifically, right inferior frontal regions modulate inhibitory control via the prefrontal–basal ganglia network^98,99^. Right temporoparietal areas seem mainly associated with shifting attention to unexpected stimuli, that is, reorienting attention^100^.

Altogether, our results of the nonlinguistic switching task suggest that the difference in behavior between the two groups (faster RTs for the CLP group) might originate from differences in early stages of cognitive control processes. That is, different cognitive processes are involved in CLP and DLP groups in response to predictable switching conditions in non-linguistic tasks. This finding is supported by the pattern of brain activation in the CLP vs DLP group during the early time window, revealing differences in ERPs. The differences between CLP and DLP bilinguals are specifically observed in the time windows prior to the processes involved in stimulus identification, change detection and distinction (N200 component). This would lead to the assumption that the difference between DLP and CLP bilinguals is not limited to language related cognitive control processes, but this difference is also seen in non-linguistic cognitive control functions.

We note that this study has some limitations that encourage further consideration. First, the spatial accuracy of EEG source estimation is controversial because these approaches have less accuracy for deep sources^101^. It is notable that scalp-recorded potentials in EEG reflect synchronous activation of neuronal populations which line up in parallel. In deep sources, especially the thalamus, the geometrical alignment of neuronal populations is not in parallel, thus electrical source estimation fails to identify deep sources accurately. We therefore need to state that the source localizations reported should be interpreted cautiously. However, using a conservative statistical approach on a high-density EEG data, this limitation should be at least partially solved (Michel et al., 2004). Second, one may assume that the effect of using two EEG recording systems may affect the EEG data. This factor has been studied previously and it has been shown that when using standard research-grade EEG systems, the data do not differ between the systems across different paradigms^102^. Third, we had a limited number of accepted trials (≥ 20 trials) for ERPs. This was led by several factors such as a limited number of initial trials (70 per condition) in order to minimize participants’ fatigue. In addition, we only included the EEG data epochs of the correct responses. Also, in order to minimize the motor artefacts caused by oral response producing, we removed EEG data epochs of the trials where the subject responded faster than 600 ms (which was set as the length of our epochs after image presentation). We need also to clarify that the power analysis suggests that going down to 20 remains adequate following the literature; several previous ERP studies on language production used this number of epochs (minimum 20 epochs)^40,42,103,104^.

To conclude, our results showed faster behavioral responses in linguistic and nonlinguistic switching tasks in CLP bilinguals than in DLP bilinguals. Electrophysiological data support our behavioral findings, suggesting differential involvement of the brain areas related to language control and cognitive control when comparing the two groups. The electrophysiological effects from 450 to 515 ms with differential involvement of the left IFG and PCC are likely related to suppression of the nontarget language and selection of the lexical representation from the target language during language switching. In the nonlinguistic switching task, early effects from 40 to 150 ms can be associated with differences between CLP and DLP bilinguals in attentional processes at early stages of response inhibition.

## Supplementary Information

Supplementary Information.
