# Supplementary material for: Language distance modulates cognitive control in bilinguals
Source: Sci Rep. 2021 Dec 16;11:24131. doi: 10.1038/s41598-021-02973-x (PMC8677725; doi:10.1038/s41598-021-02973-x)
Supplement: Supplementary file 1 — Supplementary Information. [file 41598_2021_2973_MOESM1_ESM.pptx]

## Slide 1
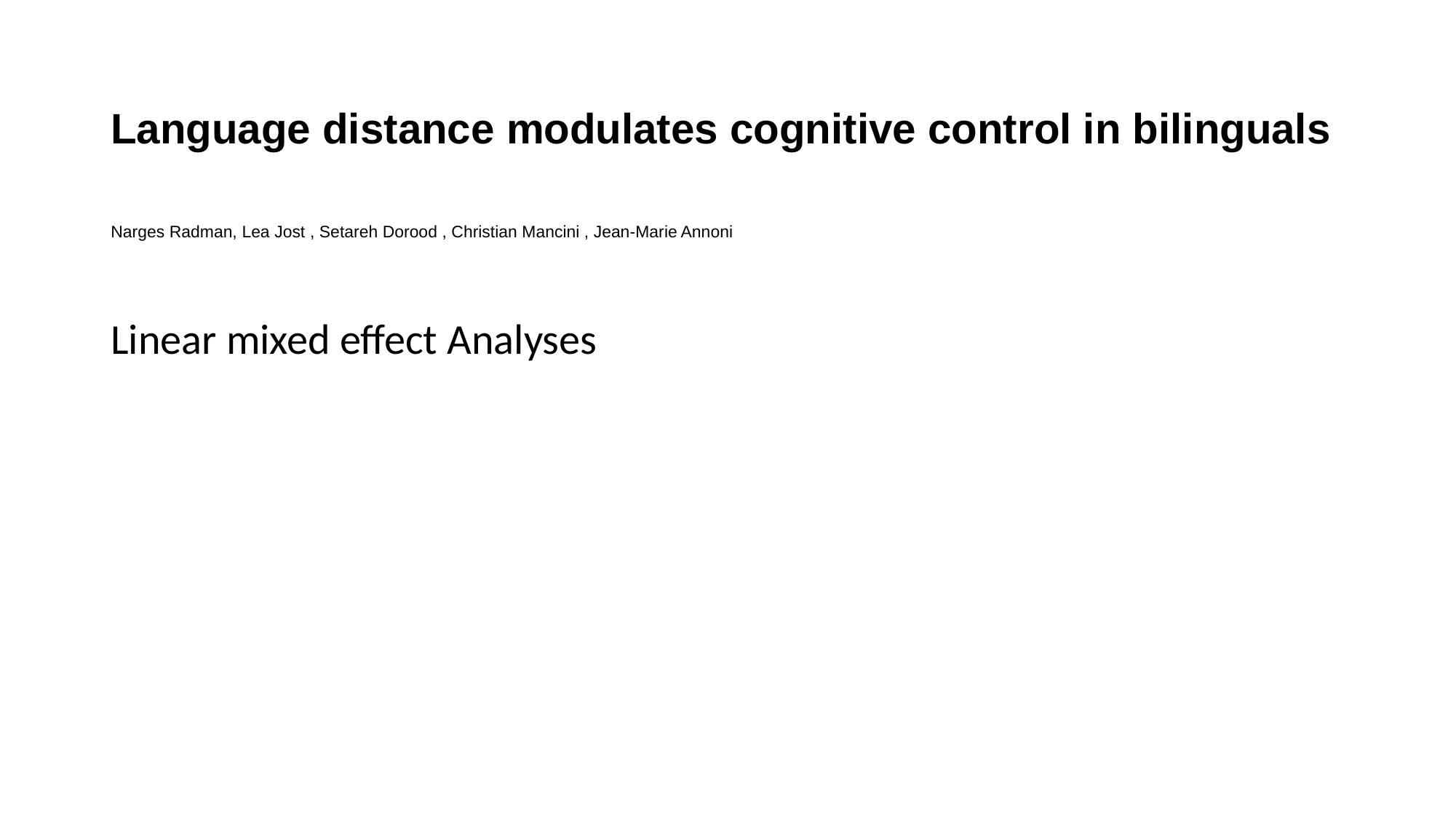

# Language distance modulates cognitive control in bilinguals
Narges Radman, Lea Jost , Setareh Dorood , Christian Mancini , Jean-Marie Annoni
Linear mixed effect Analyses

## Slide 2
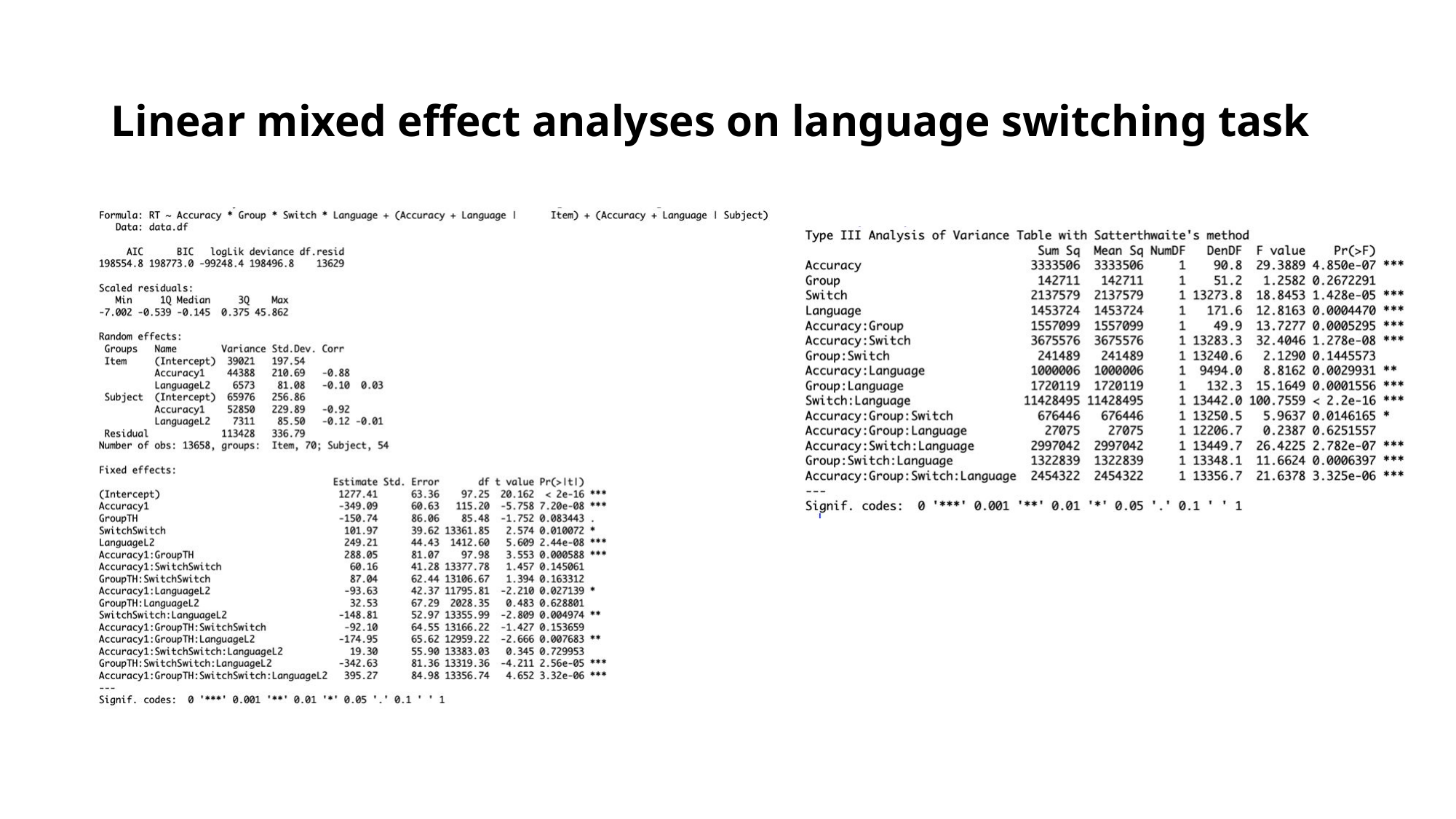

# Linear mixed effect analyses on language switching task

## Slide 3
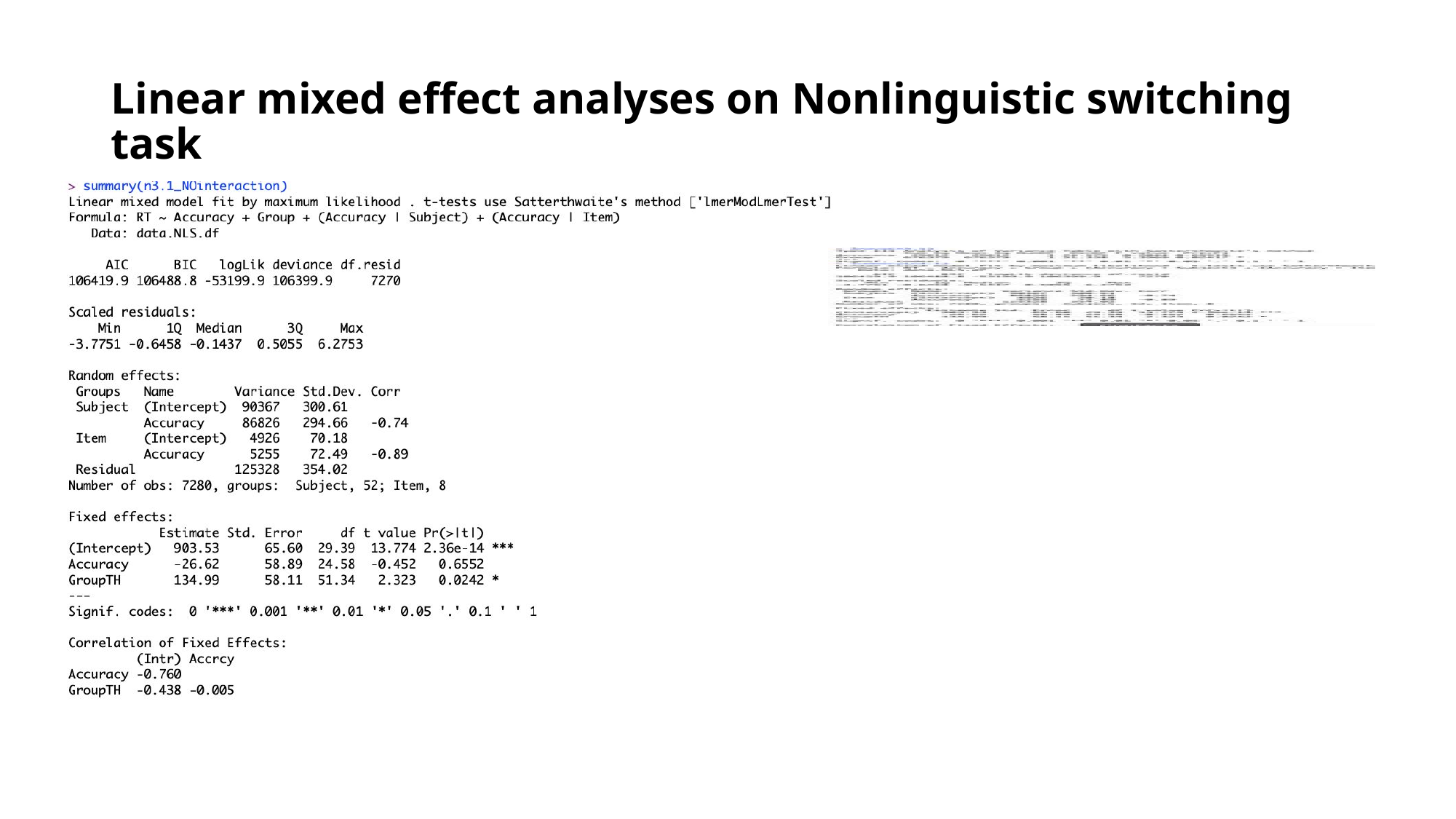

# Linear mixed effect analyses on Nonlinguistic switching task
